# Cardiogoniometry as a diagnostic tool in patients with acute coronary syndromes: results of the CGM@ACS trial

**DOI:** 10.1007/s00392-012-0452-2

**Published:** 2012-04-07

**Authors:** Ralph Tölg, Uwe Zeymer, Ralf Birkemeyer, Rainer Wessely, Holger Eggebrecht, Wolfgang Bocksch, Steffen Schneider, Gert Richardt, Christian Hamm

**Affiliations:** 1 Herzzentrum, Segeberger Kliniken GmbH, Am Kurpark 1, 23795 Bad Segeberg, Germany; 2 Med. Klinik B, Herzzentrum Ludwigshafen, Ludwigshafen, Germany; 3 Klinik für Innere Medizin III, Schwarzwald- Baar-Klinikum Villingen-Schwenningen GmbH, Villingen-Schwenningen, Germany; 4 Klinik für Kardiologie Und Angiologie, Evangelisches Bethesda-Johanniter-Klinikum Duisburg GmbH, Duisburg, Germany; 5 Cardioangiologisches Centrum Bethanien, Frankfurt, Germany; 6 Innere Medizin III, Medizinische Klinik Und Poliklinik, Tübingen, Germany; 7 Stiftung Institut für Herzinfarktforschung an der Universität Heidelberg, Ludwigshafen, Germany; 8 Herz- und Thoraxzentrum, Kerckhoff Klinik GmbH, Bad Nauheim, Germany

**Keywords:** Chest pain, Emergency, Infarction, Ischaemia, Patient management, Triage, Vector cardiography

## Abstract

**Background:**

Cardiogoniometry (CGM) is a novel electrocardiac method utilising computer-assisted three-dimensional information on cardiac potentials.

**Objective:**

To investigate the potential of CGM in discriminating non-ST-segment elevation acute coronary syndrome (NSTE-ACS) and relevant coronary stenosis upon hospital admission by prospectively comparing its sensitivity, specificity and accuracy against those of a single troponin test and a 12-lead ECG performed on admission

**Design:**

A multicenter prospective observational trial.

**Setting:**

Eight interventional cardiac centres in Germany.

**Patients:**

A cohort of 216 patients (mean age 67 years, 34.7 % female) who presented with acute chest pain or dyspnoea without ST-segment elevation and were scheduled for coronary angiography within 72 h of admission.

**Intervention:**

Pre-angiography screening by CGM, troponin test, 12-lead ECG

**Main outcome measures:**

ECG, troponin and CGM on admission compared with final diagnosis of NSTE-ACS or relevant diameter stenosis ≥70 % verified by an independent review board and an angiographic core laboratory.

**Results:**

NSTE-ACS was finally confirmed in 162 cases, whereas the remaining 54 cases without proof of NSTE-ACS served as controls. Diagnostic sensitivity for NSTE-ACS was 28, 50 and 69 % and specificity 78, 96 and 54 % for first ECG, serial troponin and first CGM, respectively. Accuracy was 40, 62 and 65 %. The sensitivity of the tests to detect relevant coronary stenosis (*n* = 126) was 32, 53 and 74 %, respectively. The sensitivity of CGM to detect NSTE-ACS (65 %) or relevant stenosis (71 %) was high even in patients with normal troponin and ECG.

**Conclusions:**

CGM can detect NSTE-ACS at first medical contact. CGM in conjunction with traditional markers, 12-lead ECG and troponin may effectively aid early decision making in patients presenting with acute chest pain.

## Introduction

Acute coronary syndrome (ACS) comprises a spectrum of presentations including unstable angina pectoris and myocardial infarctions which are further subdivided into infarctions with and without ST-segment elevations. In the majority of cases, the pathology underlying ACS results from erosion or rupture of a thin fibrous cap of a lipid-rich atherosclerotic plaque, leading to thrombus formation [1]. Patients with unstable angina exhibit ischaemic symptoms, although biomarkers reveal no evidence of myocardial necrosis [2]. Patients with clinical symptoms and elevated cardiac biomarkers may present with ST-segment elevation myocardial infarction (STEMI) or non-ST-segment elevation myocardial infarction (NSTEMI). Current guidelines recommend early reperfusion therapy for STEMI [3, 4] and an early invasive strategy for NSTEMI [5]. A fast, simple and reliable diagnostic tool is critical for optimal management of ACS patients [2, 6]. While a STEMI can be rapidly detected by ECG in most instances, NSTEMI and unstable angina, subsumed as NSTE-ACS, may not show diagnostically relevant changes on the ECG or elevations of cardiac markers at first medical contact [7]. This dilemma is responsible for a significant consumption of time and healthcare resources [8]. There is an unmet need for a practical, cost-effective and accurate diagnostic tool capable of detecting patients with NSTE-ACS or even relevant stenoses at first medical contact. Acknowledging that a chest pain unit can improve long-term outcome [21] by prompt identification and treatment of patients with an ACS this is even more requested.

Cardiogoniometry (CGM) is a novel electrodiagnostic method utilising computer-assisted three-dimensional information on cardiac potentials. It was introduced by Sanz et al. [9] and has been adjusted for detection of coronary artery disease (CAD) in recent years by Schüpbach and Hübner [10, 11]. In a prospective cohort of 332 subjects undergoing coronary angiography, the diagnostic accuracy of CGM was 71 % for detecting >50 % stenoses of the coronary arteries and thereby significantly better than that of a 12-lead ECG (*p* < 0.003) [10]. Birkemeyer et al. [18] prospectively evaluated the accuracy of CGM versus cardiac magnetic resonance imaging (MRI) in 40 patients. CGM reached a high accuracy of 83 %, a sensitivity of 70 % and a specificity of 95 %. In summary, there are CGM studies of approximately 2,000 patients in various settings versus different reference standards available. In a review based on meta analyses, the sensitivity of CGM has been described to be around 73 % and the specificity around 84 %, respectively, to detect CAD, myocardial ischaemia or structural myocardial damages [12]. We therefore sought to investigate the potential of ECG, troponin and CGM to detect patients with NSTE-ACS after hospital admission.

## Methods

### Design

This was a prospective observational trial to compare the sensitivity, specificity and accuracy of (not high-sensitive) troponin test, 12-lead ECG and CGM for detecting NSTE-ACS and/or relevant stenoses (≥70 %) on admission. Final confirmation of the diagnosis was post hoc and based on all clinical information, including serial measurements, but the reviewers were blinded to the CGM results.

### Patient selection

In the prospective CGM@ACS (*C*ardio*G*onio*M*etry for early detection of acute myocardial ischaemia in *A*cute *C*oronary *S*yndromes) study, patients admitted to one of the eight participating centres with acute chest pain and/or dyspnea were eligible for inclusion if they received an ECG and troponin test on admission and were scheduled for coronary angiography within 72 h. Exclusion criteria were: ST-segment elevation myocardial infarction (STEMI), cardiogenic shock, presence of significant cardiac ectopic beats, pacemaker, tachycardia >150 beats/min, bundle branch block and/or atrial fibrillation. All patients had to provide informed consent before inclusion and their data had to be entered anonymously in an online database. 216 patients were finally eligible for analysis.

### Validation of the clinical diagnosis

**Fig. 1:**
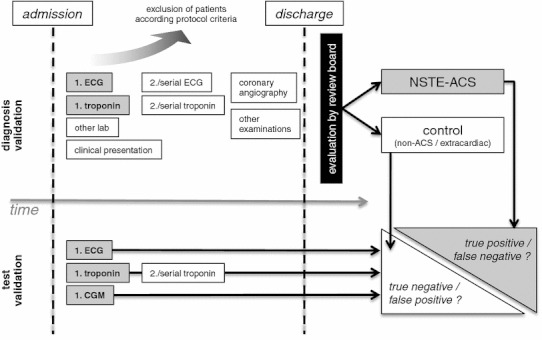
Flowchart of the 216 eligible patients analysed per protocol. The final diagnosis was validated post hoc by independent reviewers, who based their judgment on all clinical information including but not limited to serial measurements of biomarkers and ECG recordings. They were blinded to the CGM recordings. The patients were assigned to the NSTE-ACS group (*n* = 162) or control group (*n* = 54). The review board’s assignment validated how well the results of the first ECG, first or serial troponin and first CGM at the time of admission discriminated NSTE-ACS against controls

Two independent, blinded investigators (study review board) evaluated the clinical diagnosis of the patients based on the data in the online database: patient history, cardiovascular risk profile and symptoms at admission. The following parameters were available for all patients included into the study: ECG, two-dimensional and M-Mode echocardiography, troponin, CK, CK-MB, creatinine and NT-proBNP. Serial recordings of ECG and serial biomarker results were made available whenever applicable by clinical routine. The coronary angiography reports were provided by the institutions, and the investigators had access to the original angiographies. Based on this post hoc validation on the patient’s entire clinical course, including coronary angiography, patients were assigned to two clinical groups blinded by the results of CGM (Fig. 1): The first group, NSTE-ACS, subsumed NSTEMI and unstable angina. NSTEMI was defined as patients presenting with acute chest pain without persistent ST-segment elevation according to the definition provided by Thygesen et al. [13], but with elevation of myocardial markers such as troponin or CK-MB showing a typical rising and falling pattern. Unstable angina was defined according to Cannon and Braunwald [14] in patients with negative biomarkers. The second group consisted of all patients with either cardiac symptoms, but no ACS, or extracardiac symptoms; these patients served as the control group. Thus, the control group included patients with existing, but stable coronary heart disease with no acute ischaemic symptoms and patients with myocarditis or pericarditis, cardiomyopathy, valvular heart disease, hypertensive crisis, pleuritis, pneumonia, aortic syndrome, gastrointestinal disorders, such as gastritis or peptic ulcer, or musculoskeletal disorders, such as discopathy, costochondritis or muscular hardening.

All coronary angiograms were reviewed by an independent core laboratory (Institut für Herzinfarktforschung Ruhr, Essen, Germany) that graded coronary stenosis by quantitative coronary analysis. A stenosis of at least 70 % in diameter in a major coronary artery was regarded as relevant.

The trial protocol and informed consent was approved by an independent ethical review board (Ethikkommission der Landesärztekammer Rheinland-Pfalz, Mainz, Germany).

### Test validation

We compared the results of the immediate CGM, first ECG at rest and first troponin test taken at the moment of admission to the hospital with the final diagnosis established by the review board. If indicated, serial troponin tests were collected and included in the comparison. In this highly selected but well diagnosed cohort, we evaluated the true positive and false negative test results obtained in the detection of NSTE-ACS and the true negative and false positive test results used to detect patients of the control group, respectively (Fig. 1). Secondly, comparable analysis was performed with regard to relevant coronary stenoses.

### Principles of cardiogoniometry

The trigonometric principles of CGM have been published in detail elsewhere [9–12]. Briefly, four electrodes define two planes perpendicular to each other. Vectorial addition of the potentials measured between three electrodes in each plane yields a vector that corresponds to the projection of the heart vector into this plane. Using the vector projections in the two orthogonal planes, the heart vector can be reconstructed for every millisecond. The vector orientation indicates the direction and the vector length the intensity of the electrical field generated by the heart.

**Fig. 2:**
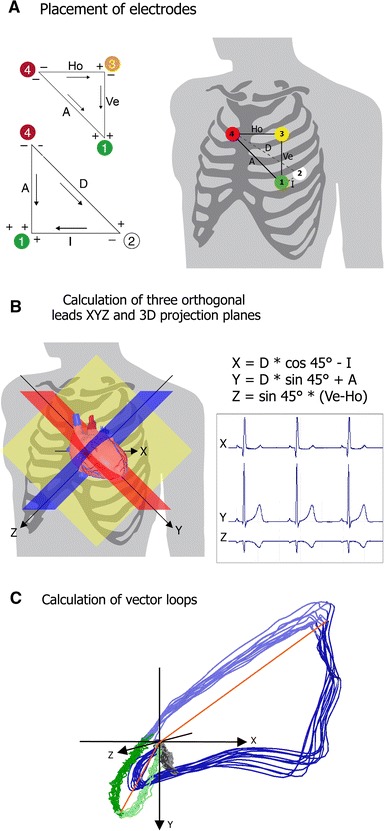
Principles of cardiogoniometry, **a** The four signal electrodes are placed on the thorax: at point 1 (*green*), equivalent to point V4 of Wilson, in the fifth intercostal space in the midclavicular line; at point 2 (*whit*e) sagittal to electrode 1 on the back (point V8 of Wilson); at point 3 (*yellow*) perpendicularly above electrode 1 at 0.7 times the distance between points 1 and 2; at point 4 (*red*) to the right of point 3 at the same distance as between points 1 and 3. The fifth electrode is ground and can be attached somewhere above the right hip. The leads are defined as: 4-2 D (dorsal), 4-1 A (anterior), 2-1 I (inferior), 4-3 Ho (horizontal), 3-1 Ve (vertical). **b** Points 4–2–1 define the oblique sagittal plane (OSP) (*red*); points 4–3–1, the frontal plane (*yellow*). The third plane (*blue*) is orthogonal to the two other planes and contains point 3; it is the sagittal plane perpendicular to the OSP. Projection *X* is orientated in an anterodorsal direction and lies in the OSP and the sagittal plane perpendicular to the OSP. Projection *Y* is orientated in a baso-apical direction and lies in the OSP (4–2–1) and the frontal plane (4–3–1). Projection *Z* is orientated in a supero-inferior direction relative to the OSP and lies in the frontal plane (4–3–1) and the sagittal plane perpendicular to the OSP. **c** Vector-loops can be calculated within a Cartesian coordinate system using *X*, *Y* and *Z* coordinates of the heart vector at each ms recording. Note the R-loops (*blue*) and T-loops (*green*) of 12 heart cycles and maximum vectors of both loops (*red*). The maximum vectors are calculated on the median loops (with kind permission from Springer Science + Business Media from [11], figure number 1)

Three mutually orthogonal projections *X*, *Y* and *Z* are calculated trigonometrically. This Cartesian coordinate system (*XYZ*) is roughly orientated according to the anatomy of the heart and its orientation in the chest, which is greatly advantageous for visualising spatial de- and repolarisation and produces an immediate cardiac interpretation of the recorded vectorial information (Fig. 2). The *X*-axis has an anteroposterior orientation (values with positive signs have a posterior location). The *Y*-axis has a left-oblique-sagittal baso-apical orientation (values with positive signs point to the apex and those with negative signs to the base of the heart). The main plane (oblique sagittal plane) is defined by the *X*- and *Y*-axes. The *Z*-axis is perpendicular to the two other planes (values with negative signs point up). The frontal plane is defined by the *Y*- and *Z*-axes. The plane defined by the *X*- and *Z*-axes is also a sagittal plane, which is perpendicular to the oblique sagittal and frontal planes and separates the apical and basal portions of the heart.

CGM differs from conventional seven-lead Frank vectorcardiography in two essential respects. Firstly, CGM is recorded with five leads (4 electrodes and 1 ground) without intercalated resistor networks (uncorrected technique). The geometrical electrode placement in an orthogonal system avoids the distortions associated with vectorcardiography techniques. In CGM, the electrode position and the trigonometrical constructions lead to a mathematically correct orthogonality of *XYZ* projections. Secondly, CGM projection planes are not aligned with the body planes but rotated to approximately match the anatomical orientation of the heart similar to the short axis scan of an MRI.

A total of 350 parameters can be extracted from the CGM data fully automatically and divided into main classes: Angles consisted of longitude and latitude angles of the P, R and T-loops, in particular, the angle of the maximum vectors and at further defined positions in the loops and angles between the maximum vectors of the P, R and T-loops. The amplitude class comprised the minima/maxima and amplitude ratios of P, R and ST/T segments. Shapes and eccentricities were used to describe the P, R and T-loops. Potential distributions covered the P, R and ST/T-loops in octants. Velocities were classified as absolute values and ratios of the P, R and T-loops. In addition, all parameters were classified according to variability (adapted from [11]). As CAD may alter the surface potential of the global cardiac activity in different ways, depending on the affected area of the myocardium, a combination of independent penalising variables has been compiled to gender and rhythm specific sets and is used in the standard CGM method since 2008. Hübner et al. proved that specific CGM parameters are significant and suitable for detecting predefined CAD categories and the systematically computed algorithm in the standard CGM method derived from a diagnostic set of stenosis pattern-specific, parallel and equally ranked parameters enables global CAD detection. Although not every parameter is useful for every CAD category, the overall parameter set inside the standard CGM algorithm is independent of coronary stenosis localisation and distribution or ischaemia pattern [11]. All parameters of a set must be in their reference range to define a score of zero (normal CGM). For each parameter outside of its reference range a negative score point is counted [10, 11]. Any negative score point reflects a pathologic (positive) CGM. In summary, CGM captures the temporospatial informations and their beat-to-beat variance breaking them down to several relevant and measurable parameters that can be taken as reference. In contrast, conventional ECG only takes into account the cardiac electricity at 12 lead points over one heart cycle.

### Cardiogoniometry measurements

The CGM measurements were obtained by placing five leads on the supine patient and using commercially available hard- and software (Cardiologic Explorer, enverdis, Jena, Germany). During the 12-s recording, the patient was asked to perform shallow breathing to keep thoracic excursions to a minimum.

### Interpreting a CGM finding

When all parameters recorded on a patient are within normal range, a score of zero is produced, i.e., the CGM is negative. If any parameter is out of range, the score is below zero, thus defining a pathological or “positive” CGM. The immediate, automatically produced finding was registered as the CGM diagnosis, which was entered in the electronic case record form. Albeit the phrase “score” is used, the CGM produces a dichotomic result of “positive” or “negative” CGM. The numerical value of the score below zero does not reflect a measure of extent or severity of ischaemia.

### Troponin and ECG

Troponin was measured as T or I according to the local practice of each site. No highly sensitive troponin was used. A value within the reference limit of the respective test kit was regarded as negative, whilst a value above the upper limit of normal was regarded as positive. With respect to serial troponin results, a test was regarded positive if at least one of the measurements was positive. Conversely, a serial troponin testing had to be regarded negative if every single test at different time points was negative. An ECG with persistent or transient ST-segment, new horizontal or down-sloping ST depression ≥0.05 mV in two contiguous leads and/or T inversion ≥0.1 mV in two contiguous leads with prominent R-wave or R/S ratio >1 was regarded as indicative of acute myocardial ischaemia and therefore registered as positive; all other cases were registered as negative[13].

### Statistical analysis

Data are presented as absolute numbers, percentage or medians as appropriate. Categorical values and the predictive values were compared by Chi-square test or Fisher’s exact test in smaller sample sizes. Continuous variables were compared using the two-tailed Wilcoxon rank sum test. The McNemar test was performed to compare sensitivities, specificities and the diagnostic accuracy of CGM ECG, and Troponin. *p* values <0.05 were considered significant. All statistical analyses were performed using SAS statistical analysis software 9.1 (Cary, North Carolina).

## Results

In the total cohort of 216 patients, NSTE-ACS was detected in 162 patients, the remaining 54 patients were diagnosed to have cardiac disease, but not ACS (*n* = 20), or to have chest pain of extracardiac origin (*n* = 34) after post hoc verification of the diagnosis by the study review board.

The baseline characteristics of the patients in each group are shown in Table 1.

**Table 1:** Baseline characteristics and angiographic details

	NSTE-ACS	Control	*p* value
*n*	162	54	
Age (years)	69.5 (59.0–75.0)	59.0 (51.0–70.0)	<0.001
Female	32.7 %	40.7 %	0.28
History			
Diabetes	28.4 %	14.8 %	<0.05
Hyperlipidemia	67.9 %	46.3 %	<0.01
Arterial hypertension	88.9 %	72.2 %	<0.01
Family history of CAD	27.2 %	16.7 %	0.12
Renal insufficiency	10.5 %	1.9 %	<0.05
Smoker	27.8 %	35.2 %	0.30
Previous MI	27.8 %	9.3 %	<0.01
Previous CABG	16.7 %	0 %	<0.01
Previous PCI	42.0 %	16.7 %	<0.001
Previous Stroke	6.2 %	5.6 %	0.87
Peripheral artery disease	4.9 %	0 %	0.10
Clinical presentation at admission			
Duration of symptoms (h:min)	10:56 (2:48–50:47)	6:31 (2:20–41:09)	0.29
Chest pain	99.4 %	100 %	0.56
Dyspnea	16.7 %	7.4 %	0.09
Systolic blood pressure (mmHg)	140 (122–160)	140 (127–150)	0.42
Diastolic blood pressure (mmHg)	80 (70–89)	78 (70–80)	0.42
Heart rate (bpm)	71 (63–81)	67 (62–75)	0.15
Killip class 1 + 2	98.8 %	100 %	1.00
Medication at admission			
ASA	71.6 %	68.5 %	0.67
Thienopyridines	24.1 %	14.9 %	0.15
Anticoagulation	18.6 %	18.5 %	1.00
Betablocker	60.5 %	51.9 %	0.26
Statins	42.6 %	29.6 %	0.09
ACE inhibitors or ARB or renin inhibitors	53.1 %	48.1 %	0.53
Angiographic details (core lab)			
Door-to-needle time (h:min)	9:00 (3:27–24:28)	20:30 (4:49–27:31)	<0.05
No CAD detected	6.9 %	85.2 %	<0.0001
1-coronary vessel disease	32.1 %	14.8 %	<0.05
2- and 3- coronary vessel disease	61.0 %	0 %	<0.0001
Stenosis ≥70 %	80.3 %	0 %	<0.0001
Impaired TIMI flow (<3)	45.3 %	0 %	<0.0001
PCI performed	60.4 %	0 %	<0.0001
EF < 40 %	4.3 %	2.9 %	1.00
EF > 55 %	70.5 %	91.4 %	<0.05

Values in percent or median with interquartile range in brackets. Groups NSTE-ACS and control (patients with non-ACS or extracardiac disease) as defined in the text under methods

*MI* myocardial infarction, *CABG* coronary artery bypass graft, *PCI* percutaneous coronary intervention, *CAD* coronary artery disease, *ASA* acetyl salicylic acid, *ACE* angiotensin converting enzyme, *ARB* Angiotensin receptor blocker,* EF* ejection fraction

Patients presenting with NSTE-ACS were on average >10 years older than the controls and had a higher rate of diabetes mellitus (28.4 %). Cardiovascular risk factors like hyperlipidemia, arterial hypertension, family history were more common in NSTE-ACS patients. However, smoking was equally present in both groups. A previous history of coronary and peripheral artery disease was more often present in NSTE-ACS patients, while prior cerebrovascular events were equally distributed in both groups.

Patients who later turned out to have NSTE-ACS had a longer history of symptoms prior to hospital admission than the control group, however, this was not significant. In contrast, the time from admission to coronary angiography (door-to-needle time) was significantly shorter in NSTE-ACS patients. The clinical presentation and haemodynamics of patients with NSTE-ACS were similar to those of the control group, although there tended to be more concomitant dyspnea in the NSTE-ACS group.

Both groups did not differ in their medication at admission, with a tendency towards a higher use of statins in the NSTE-ACS group. The control group also showed a high intake of acetyl salicylic acid (ASA; 68.5 %), beta-blockers (51.9 %) and blockers of the renin–aldosterone–angiotensin system (48.1 %), suggesting the presence of cardiovascular disease. The use of thienopyridines (14.9 %) in the control group was in line with the rate of previous PCI (16.7 %), as shown in Table 1.

The angiographic details of the core laboratory analysis are given in Table 1.

The diagnostic yield of the different methods in detecting NSTE-ACS versus control and in detecting relevant coronary stenoses (≥70 % diameter) is shown in Table 2.

**Table 2:** Diagnostic yield of method in detecting patients with NSTE-ACS and with relevant coronary stenoses

	ECG	First troponin	Serial troponin	CGM
Detection of NSTE-ACS				
Sensitivity	28 % (45/162)*	34 % (55/162)*	50 % (81/162)*	69 % (111/162)
Specificity	78 % (42/54)^‡^	98 % (53/54)*	96 % (52/54)*	54 % (29/54)
PPV	79 % (45/57)^#^	98 % (55/56)^†^	98 % (81/83)*	82 % (111/136)
NPV	26 % (42/159)^#^	33 % (53/160)^#^	39 % (52/133)^#^	36 % (29/80)
Accuracy	40 % (87/216)*	50 % (108/216)*	62 % (133/216)^#^	65 % (140/216)
Detection of relevant coronary stenoses				
Sensitivity	32 % (40/126)*		53 % (67/126)*	74 % (93/126)
Specificity	80 % (68/85)*		81 % (69/85)*	51 % (43/85)
PPV	70 % (40/57)^#^		81 % (67/83)^#^	69 % (93/135)
NPV	44 % (68/154)^#^		54 % (69/128)^#^	57 % (43/76)
Accuracy	51 % (108/211)^†^		64 % (136/211)^#^	64 % (136/211)

Serial troponin reflects any troponin from first up to third measurement, if indicated

*PPV* positive predictive value, *NPV* negative predictive value

* *p* < 0.001 compared to CGM

^†^ *p* = 0.001 compared to CGM

^‡^ *p* = 0.002 compared to CGM

^#^ Not significantly different (*p* > 0.05) compared to CGM

In our cohort of patients presenting with acute chest pain, half (*n* = 106) had a negative ECG and a negative troponin. In this subgroup, CGM revealed a sensitivity of 65 % and accuracy of 66 % in detecting NSTE-ACS and a sensitivity of 71 % and accuracy of 63 % in detecting patients with significant coronary stenoses in the coronary angiogram (Table 3).

**Table 3:** Value of CGM in patients with negative ECG and negative serial troponin (*n* = 106)

	NSTE-ACS	Stenosis ≥70 %
Sensitivity	65 % (42/65)	71 % (34/48)
Specificity	59 % (24/41)	58 % (31/53)
PPV	71 % (46/59)	61 % (34/56)
NPV	51 % (24/47)	68 % (31/45)
Accuracy	66 % (70/106)	63 % (65/101)

All test evaluations refer to first measurement or recording

*PPV* positive predictive value, *NPV* negative predictive value

## Discussion

CGM is a novel electrodiagnostic and vectorcardiographic tool proven to detect myocardial ischaemia and CAD [9–12]. The classic vectorcardiography, a forerunner of CGM, could not be implemented into routine clinical practice because it was too difficult to record and interpret. As a major advancement over the old method, state-of-the-art CGM fully automatically generates a simple and readily available diagnosis based on a combination of temporal and spatial parameters and their variabilities [11]. Thus, CGM delivers more cardio-electric information than a conventional 12-lead ECG.

The principal finding of this study is that CGM proved more sensitivity in detecting NSTE-ACS and relevant coronary stenosis than conventional ECG or a non-high-sensitive troponin as a screening method performed at first medical contact. The major limitations of the well-established troponin test are that it is dependent on time-relevant protein release kinetics and the fact that, by definition, patients with unstable angina cannot be diagnosed by troponin. Using conventional assays, the sensitivity of troponin T for myocardial infarction increases from 25–65 % at hospital admission to 59–90 % after 2–6 h [15–17] and reaches almost 100 % after 6–12 h [15, 16]. These numbers are somewhat lower at admission when troponin I is used, but almost similar to troponin T later on [15–17]. As our cohort not only comprised patients with myocardial infarction but also patients with unstable angina, the sensitivity of the troponin test could, by definition, maximally approach the ratio of infarction patients. On the other hand, the subgroup of NSTEMI is defined by elevation of myocardial markers, and false positive troponin due to other causes is very rare. Therefore, the positive predictive value of troponin has to be high, which only means if there is a positive troponin there is a very high chance for the diagnosis of NSTE-ACS. Notably, the negative predictive values of troponin and CGM are almost at the same level, so the chance to detect a true “healthy” subject is comparable. Hence, the CGM tool produced a high sensitivity and accuracy for the early discrimination of NSTE-ACS in the overall cohort. Moreover, CGM results were acquired and available immediately at the time of admission and their sensitivity did not show any time dependency. CGM should neither challenge the role and clinical importance of cardiac markers like troponin, nor does it provide any information on outcome so far, but with its sensitivity for NSTE-ACS it could be a complementary diagnostic tool in the first assessment of patients, especially in early presenters after chest pain, as the first ECG and troponin at admission showed a very poor sensitivity of 28 and 34 %, respectively. Undoubtedly, this does not necessarily mean that all positive CGM should be answered by an immediate coronary angiogram but could at least focus the physicians’ attention especially in the case of a normal ECG and normal troponin at admission. Overall, performance of CGM was at least superior to conventional 12-lead ECG. Of course, a significantly higher sensitivity is contrasted by a significantly lower specificity of CGM compared to ECG, but CGM shows higher positive and negative predictive values and a better accuracy than ECG.

**Fig. 3:**
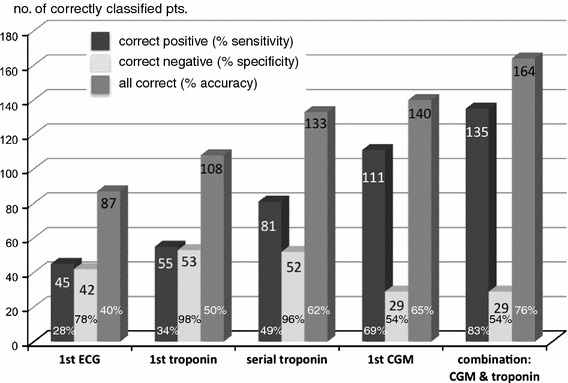
Numbers of correctly positively classified patients out of 162 patients with NSTE-ACS based on the first ECG, the first troponin, all consecutive (serial) troponin measurements, the first CGM and a combination of the first CGM and first troponin results are shown as *dark*-*grey bars* (percentage reflects sensitivity). The numbers of correctly negatively classified patients in the 54 controls are shown in *light*-*grey bars* (percentage reflects specificity). The numbers for all 216 patients correctly screened (positive and negative) are represented by *medium*-*grey bars* (percentage reflects accuracy)

In our study setting, where clinical decision making was blinded to the results of the CGM recording, we observed that an average delay of between 9 and 24 h elapsed before coronary angiography and intervention, even in the group presenting with NSTE-ACS. This “early invasive strategy” consumed significant clinical resources before final therapy was initiated. Of course, the prolonged door-to-needle time might also have been due to purely logistical reasons like admission in the late afternoon or evening with no need for an emergent catheter lab procedure. We do not want to question the standard of care afforded by serial troponin and ECG recordings; nevertheless, CGM can indeed speed up decision making or help to triage patients into chest pain units at least in pre-hospital setting where troponin tests may not be available. Finally, the combined use of a positive CGM and/or a positive troponin test would increase sensitivity for NSTE-ACS to 83 % in cohorts like ours (Fig. 3).

In this real-life scenario, it is striking that barely half of the patients (*n* = 106) scheduled for coronary angiography based on the clinical suspicion of ACS had a negative ECG and/or a negative troponin test at first medical contact, although final diagnosis of ACS was retrospectively confirmed in almost three quarters of the cohort.

In the subgroup of patients with no abnormalities on initial ECG or troponin test, CGM maintained a consistently high sensitivity of 65 and 71 % for detecting NSTE-ACS and relevant coronary stenoses ≥70 % in diameter, respectively. Proposing an algorithm, where patients would undergo an early invasive strategy, (1) if at least troponin or ECG is positive and (2) only if both are negative (*n* = 106 in this setting), then CGM would add for decision making, so one would have detected additional 42 patients with NSTE-ACS by positive CGM out of this latter group (2) (number needed to treat = 2.5). Conversely, 17 patients of these 106 would have undergone an early coronary angiography but without final confirmation of NSTE-ACS (number needed to harm = 6.2).

Based on these findings, CGM may be used as a complementary tool to the tests currently recommended by the guidelines to early discriminate patients with ACSs from patients with non-cardiac chest pain. This advantage would be especially pertinent to outpatient settings.

In our study cohort, CGM’s specificity proved lower than that of ECG and troponin, which might have partially been because CGM algorithms are calibrated to detect ischaemia along with myocardial scars and structural myocardial lesions. Accordingly, the algorithms do not discriminate acute ischaemia and chronic disease from prior coronary events. Also, there was a certain prevalence of patients with a previous history of myocardial infarction and coronary interventions in the subgroup of patients who were not classified with NSTE-ACS retrospectively. Birkemeyer et al. [18] prospectively evaluated the accuracy of CGM versus stress cardiac MRI, considered the non-invasive gold standard of ischaemia diagnosis. In their unselected cohort of 40 patients, CGM findings were compared against pathological perfusion and/or the presence of late enhancement (20 patients in total) during cardiac MRI. CGM achieved a total accuracy of 83 %, i.e., a sensitivity of 70 % and a specificity of 95 %, whilst its positive predictive value was 93 % [18]. These findings underscore the hypothesis that the moderately lower specificity within the CGM@ACS trial was mainly caused by the high prevalence of stable CAD and structural myocardial lesions in the control cohort.

The major limitation of the study was that we investigated a highly selected cohort of patients with chest pain who underwent an early invasive treatment strategy. We therefore might have a higher pre-test probability for NSTE-ACS patients resulting in a larger number of patients in the final NSTE-ACS group and fewer patients in the control group. This presumption would be in line with the high rate of patients showing a history of CAD such as previous myocardial infarction or previous revascularisation procedures as shown in Table 1. Even the control group comprises a cohort of patients with relevant CAD or prior myocardial damage—nearly one-sixth of them had had a previous PCI and nearly one-tenth a previous myocardial infarction (Table 1). Therefore, our patient population is not representative of patients with chest pain seen in an outpatient scenario. Secondly, the broader use of highly sensitive cardiac troponin—compared to that used in this study—would detect more NSTEMIs [19, 22, 23]. As these next-generation assays can measure up to tenfold lower troponin concentrations than conventional assays [20], we might have obtained higher sensitivity levels if we had used such assays in this study cohort. Novel biomarkers for early diagnosis of ACS like fatty acid-binding protein [24], ischaemia-modified-albumin [25] or copeptin [26] may be promising, but they could not yet demonstrate incremental value especially over high-sensitive troponin. Thirdly, interpretation by the independent review board was solely based on written documentation and written assessment of the patient’s clinical presentation and not on a direct examination or interrogation. Last, the sample size of this study was still low, so a validation of these findings in larger collectives or a more general population is needed. The data should be therefore interpreted as hypothesis generating but not proof of additional value of CGM in the routine chest pain unit care so far.

In conclusion, CGM is a novel, easy-to-use electrodiagnostic method that can help screen patients with acute chest pain at first medical contact. In cohorts presenting with chest pain, CGM demonstrated high diagnostic sensitivity and accuracy in detecting patients with NSTE-ACS or relevant coronary stenoses. Given CGM’s comparably high sensitivity and accuracy in cases where both troponin and ECG are negative, it may provide added benefit as a diagnostic tool in the early detection of ACSs.

## References

[1] Hansson GK (2005). Inflammation, atherosclerosis, and coronary artery disease. N Engl J Med.

[2] Anderson JL, Adams CD, Antman EM, American College of Cardiology; American Heart Association Task Force on practice guidelines (Writing Committee to revise the 2002 guidelines for the management of patients with unstable angina/Non-ST-elevation myocardial infarction); American College of Emergency Physicians; Society for Cardiovascular Angiography and Interventions; Society of Thoracic Surgeons; American Association of Cardiovascular and Pulmonary Rehabilitation; Society for Academic Emergency Medicine (2007). ACC/AHA 2007 guidelines for the management of patients with unstable angina/non-ST-elevation myocardial infarction: a report of the American College of Cardiology/American Heart Association Task Force on Practice Guidelines (Writing Committee to Revise the 2002 Guidelines for the Management of Patients With Unstable Angina/Non-ST-Elevation Myocardial Infarction) developed in collaboration with the American College of Emergency Physicians, the Society for Cardiovascular Angiography and Interventions, and the Society of Thoracic Surgeons endorsed by the American Association of Cardiovascular and Pulmonary Rehabilitation and the Society for Academic Emergency Medicine. J Am Coll Cardiol.

[3] Weaver WD, Simes RJ, Betriu A (1997). Comparison of primary coronary angioplasty and intravenous thrombolytic therapy for acute myocardial infarction: a quantitative review. JAMA.

[4] Keeley EC, Boura JA, Grines CL (2003). Primary angioplasty versus intravenous thrombolytic therapy for acute myocardial infarction: a quantitative review of 23 randomised trials. Lancet.

[5] Cannon CP, Weintraub WS, Demopoulos LA (2001). TACTICS (Treat Angina with Aggrastat and Determine Cost of Therapy with an Invasive or Conservative Strategy)—thrombolysis in myocardial infarction 18 investigators. Comparison of early invasive and conservative strategies in patients with unstable coronary syndromes treated with the glycoprotein IIb/IIIa inhibitor tirofiban. N Engl J Med.

[6] Canadian Cardiovascular Society; American Academy of Family Physicians; American College of Cardiology (2008). 2007 focused update of the ACC/AHA 2004 guidelines for the management of patients with ST-elevation myocardial infarction: a report of the American College of Cardiology/American Heart Association Task Force on Practice Guidelines. J Am Coll Cardiol.

[7] Drew BJ, Pelter MM, Lee E (2005). Designing prehospital ECG systems for acute coronary syndromes. Lessons learned from clinical trials involving 12-lead ST-segment monitoring. J Electrocardiol.

[8] Forberg JL, Henriksen LS, Edenbrandt L (2006). Direct hospital costs of chest pain patients attending the emergency department: a retrospective study. BMC Emerg Med.

[9] Sanz E, Steger JP, Thie W (1983). Cardiogoniometry. Clin Cardiol.

[10] Schüpbach WM, Emese B, Loretan P (2008). Non-invasive diagnosis of coronary artery disease using cardiogoniometry performed at rest. Swiss Med Wkly.

[11] Hübner T, Schüpbach WM, Seeck A (2010). Cardiogoniometric parameters for detection of coronary artery disease at rest as a function of stenosis localization and distribution. Med Biol Eng Comput.

[12] Hübner T, Görnig M, Schüpbach M (2010). Electrocardiologic and related methods of non-invasive detection and risk stratification in myocardial ischemia: state of the art and perspectives. Ger Med Sci.

[13] Thygesen K, Alpert JS, White HD (2007). Universal definition of myocardial infarction. J Am Coll Cardiol.

[14] Cannon CP, Braunwald E, Libby P, Bonow RO, Mann DL, Zipes DP, Braunwald E (2008). Unstable angina and non-ST elevation myocardial infarction. Braunwald’s Heart Disease. A textbook of cardiovascular medicine, Saunders.

[15] Panteghini M, Pagani F, Bonetti G (1999). The sensitivity of cardiac markers: an evidence-based approach. Clin Chem Lab Med.

[16] Tucker JF, Collins RA, Anderson AJ (1997). Early diagnostic efficiency of cardiac troponin I and Troponin T for acute myocardial infarction. Acad Emerg Med.

[17] Balk EM, Ioannidis JP, Salem D (2001). Accuracy of biomarkers to diagnose acute cardiac ischemia in the emergency department: a meta-analysis. Ann Emerg Med.

[18] Birkemeyer R, Jäckle S, Hajredini B et al (2011) Direct comparison of cardiogoniometry with perfusion cardiac magnetic resonance and late gadolinium enhancement. Clin Res Cardiol 100(Suppl 1):P1383 10.1093/europace/eus218 22791298

[19] Wilson SR, Sabatine MS, Braunwald E (2009). Detection of myocardial injury in patients with unstable angina using a novel nanoparticle cardiac troponin I assay: observations from the PROTECT-TIMI 30 Trial. Am Heart J.

[20] Tate JR (2008). Troponin revisited 2008: assay performance. Clin Chem Lab Med.

[21] Keller T, Post F, Tzikas S, Schneider A, Arnolds S, Scheiba O, Blankenberg S, Münzel T, Genth-Zotz S (2010). Improved outcome in acute coronary syndrome by establishing a chest pain unit. Clin Res Cardiol.

[22] Celik S, Giannitsis E, Wollert KC, Schwöbel K, Lossnitzer D, Hilbel T, Lehrke S, Zdunek D, Hess A, Januzzi JL, Katus HA (2011). Cardiac troponin T concentrations above the 99th percentile value as measured by a new high-sensitivity assay predict long-term prognosis in patients with acute coronary syndromes undergoing routine early invasive strategy. Clin Res Cardiol.

[23] Kurz K, Giannitsis E, Becker M, Hess G, Zdunek D, Katus HA (2011). Comparison of the new high sensitive cardiac troponin T with myoglobin, h-FABP and cTnT for early identification of myocardial necrosis in the acute coronary syndrome. Clin Res Cardiol.

[24] Viswanathan K, Kilcullen N, Morrell C, Thistlethwaite SJ, Sivananthan MU, Hassan TB, Barth JH, Hall AS (2010). Heart-type fatty acid-binding protein predicts long-term mortality and re-infarction in consecutive patients with suspected acute coronary syndrome who are troponin-negative. J Am Coll Cardiol.

[25] Van Belle E, Dallongeville J, Vicaut E, Degrandsart A, Baulac C, Montalescot G (2010). Ischemia-modified albumin levels predict long-term outcome in patients with acute myocardial infarction. The French Nationwide OPERA study. Am Heart J.

[26] Reichlin T, Hochholzer W, Stelzig C, Laule K, Freidank H, Morgenthaler NG, Bergmann A, Potocki M, Noveanu M, Breidthardt T, Christ A, Boldanova T, Merki R, Schaub N, Bingisser R, Christ M, Mueller C (2009). Incremental value of copeptin for rapid rule out of acute myocardial infarction. J Am Coll Cardiol.

